# 2-Hy­droxy-3-nitro­benzaldehyde

**DOI:** 10.1107/S1600536810025110

**Published:** 2010-07-03

**Authors:** Bei Tang, Guangying Chen, Xiaoping Song, Changchun Cen, Changri Han

**Affiliations:** a Key Laboratory of Tropical Medicinal Plant Chemistry of the Ministry of Education, Hainan Normal University, College of Chemistry & Chemical Engineering, Hainan Normal University, Haikou 571158, People’s Republic of China

## Abstract

The title compound, C_7_H_5_NO_4_, isolated from the leaves of *Actephila merrilliana*, is essentially planar (r.m.s. deviation = 0.026 Å). The conformation is supported by an intra­molecular O—H⋯O hydrogen bond, which generates an *S*(6) ring. In the crystal, C—H⋯O inter­actions and aromatic π–π stacking [centroid–centroid distance = 3.754 (4) Å] help to establish the packing.

## Related literature

For medicinal background, see: Ovenden *et al.* (2001 ▶); Song *et al.* (2007 ▶). For related structures, see: Rizal *et al.* (2008 ▶); Garden *et al.* (2004 ▶).
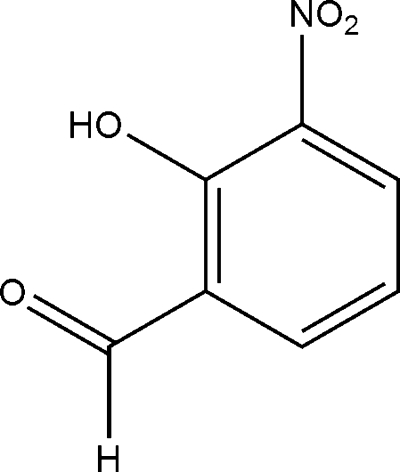


## Experimental

### 

#### Crystal data

C_7_H_5_NO_4_
*M* *_r_* = 167.12
Monoclinic, 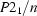

*a* = 8.8276 (7) Å
*b* = 8.7296 (8) Å
*c* = 9.011 (9) Å
β = 90.124 (1)°
*V* = 694.4 (7) Å^3^
*Z* = 4
Mo *K*α radiation
μ = 0.13 mm^−1^
*T* = 298 K
0.48 × 0.48 × 0.42 mm

#### Data collection

Bruker SMART CCD diffractometer
3289 measured reflections
1230 independent reflections
929 reflections with *I* > 2σ(*I*)
*R*_int_ = 0.026

#### Refinement

*R*[*F* ^2^ > 2σ(*F* ^2^)] = 0.038
*wR*(*F* ^2^) = 0.119
*S* = 1.07
1230 reflections
109 parameters
H-atom parameters constrained
Δρ_max_ = 0.16 e Å^−3^
Δρ_min_ = −0.18 e Å^−3^

### 

Data collection: *SMART* (Bruker, 2003 ▶); cell refinement: *SAINT* (Bruker, 2003 ▶); data reduction: *SAINT*; program(s) used to solve structure: *SHELXS97* (Sheldrick, 2008 ▶); program(s) used to refine structure: *SHELXL97* (Sheldrick, 2008 ▶); molecular graphics: *SHELXTL* (Sheldrick, 2008 ▶); software used to prepare material for publication: *SHELXTL*.

## Supplementary Material

Crystal structure: contains datablocks global, I. DOI: 10.1107/S1600536810025110/hb5495sup1.cif

Structure factors: contains datablocks I. DOI: 10.1107/S1600536810025110/hb5495Isup2.hkl

Additional supplementary materials:  crystallographic information; 3D view; checkCIF report

## Figures and Tables

**Table 1:** Hydrogen-bond geometry (Å, °)

*D*—H⋯*A*	*D*—H	H⋯*A*	*D*⋯*A*	*D*—H⋯*A*
O2—H2⋯O1	0.82	1.86	2.597 (3)	148
C5—H5⋯O2^i^	0.93	2.51	3.422 (4)	168

Symmetry code: (i) 
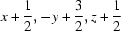
.

**Fig. 1.**
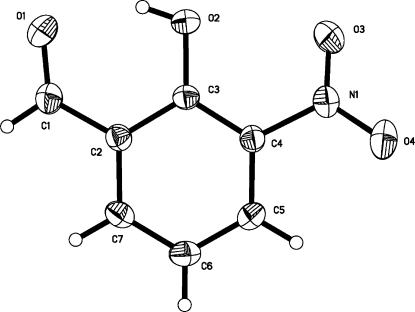
View of (I) with displacement ellipsoids drawn at the 30% probability level.

**Fig. 2.**
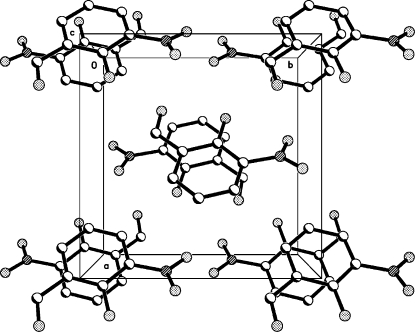
A view of the molecular packing.

## supplementary crystallographic information

### Comment

Although chemical investigations into specimens of the plant family Euphorbiaceae are very common, there have been no related reports on chemical constituents of *Actephila merrilliana* (Ovenden *et al.*, 2001). The plants in this family were used in folk medicine such as, for the treatment of hemorrhoids and as anti-inflammatory agents (Song *et al.*, 2007). The title compound was isolated from the 75% EtOH extract of the leaves of *Actephila merrilliana* which were collected from Sanya City, Hainan Province, P. R. China. We have undertaken the X-ray crystal structure analysis of the title compound in order to establish its molecular structure and relative stereochemistry. In the title compound, the bond lengths and angles in (I) have normal values, and are comparable with those in the related structures (Rizal *et al.*, 2008; Garden *et al.*, 2004). An intramolecular O—H···O hydrogen bond helps to establish an essentially planar conformation for the molecule (r.m.s. deviation = 0.0258 Å).

In the crystal, molecules are linked by intermolecular C–H···O hydrogen bonds into chains (Fig.2). The hydrogen bonds and angles are listed in Table 1.

### Experimental

Air-dried leaves of Actephila merrilliana (14.0 kg) were ground and percolated (3 × 3 h) with 75% EtOH at 60°C, which was suspended in 6 L water and then partitioned with petroleum ether, chloroform, ethyl acetate and n-BuOH, successively, yielding a petroleum ether extract, a chloroform extract, an ethyl acetate extract and a n-BuOH extract, respectively. The chloroform extract was subjected to a silica gel CC column using petroleum ether as first eluent and then increasing the polarity with EtOAc, to afford 23 fractions (A—W). Fraction M was further separated by column chromatography with a gradient of petroleum ether-EtOAc to give the title compound. The crude product was dissolved in a small amount of chloroform to obtain colourless blocks of (I) by slow evaporation of a chloroform solution at 298 K.

### Refinement

H atoms bonded to C atoms were palced in geometrically calculated position and were refined using a riding model, with *U*_iso_(H) = 1.2 *U*_eq_(C). H atoms attached to O atoms were found in a difference Fourier synthesis and were refined using a riding model, with the O—H distances fixed as initially found and with *U*_iso_(H) values set at 1.5 *U*eq(O).

### Crystal data

**Table:**

C_7_H_5_NO_4_	*F*(000) = 344
*M**_r_* = 167.12	*D*_x_ = 1.599 Mg m^−^^3^
Monoclinic, *P*2_1_/*n*	Mo *K*α radiation, λ = 0.71073 Å
Hall symbol: -P 2yn	Cell parameters from 1493 reflections
*a* = 8.8276 (7) Å	θ = 2.3–27.7°
*b* = 8.7296 (8) Å	µ = 0.13 mm^−^^1^
*c* = 9.011 (9) Å	*T* = 298 K
β = 90.124 (1)°	Block, colourless
*V* = 694.4 (7) Å^3^	0.48 × 0.48 × 0.42 mm
*Z* = 4	

### Data collection

**Table:**

Bruker SMART CCD diffractometer	929 reflections with *I* > 2σ(*I*)
Radiation source: fine-focus sealed tube	*R*_int_ = 0.026
graphite	θ_max_ = 25.0°, θ_min_ = 3.2°
phi and ω scans	*h* = −6→10
3289 measured reflections	*k* = −9→10
1230 independent reflections	*l* = −10→10

### Refinement

**Table:**

Refinement on *F*^2^	Primary atom site location: structure-invariant direct methods
Least-squares matrix: full	Secondary atom site location: difference Fourier map
*R*[*F*^2^ > 2σ(*F*^2^)] = 0.038	Hydrogen site location: inferred from neighbouring sites
*wR*(*F*^2^) = 0.119	H-atom parameters constrained
*S* = 1.07	*w* = 1/[σ^2^(*F*_o_^2^) + (0.0617*P*)^2^ + 0.1126*P*] where *P* = (*F*_o_^2^ + 2*F*_c_^2^)/3
1230 reflections	(Δ/σ)_max_ < 0.001
109 parameters	Δρ_max_ = 0.16 e Å^−^^3^
0 restraints	Δρ_min_ = −0.17 e Å^−^^3^

### Special details

**Table:**

Geometry. All e.s.d.'s (except the e.s.d. in the dihedral angle between two l.s. planes) are estimated using the full covariance matrix. The cell e.s.d.'s are taken into account individually in the estimation of e.s.d.'s in distances, angles and torsion angles; correlations between e.s.d.'s in cell parameters are only used when they are defined by crystal symmetry. An approximate (isotropic) treatment of cell e.s.d.'s is used for estimating e.s.d.'s involving l.s. planes.
Refinement. Refinement of *F*^2^ against ALL reflections. The weighted *R*-factor *wR* and goodness of fit *S* are based on *F*^2^, conventional *R*-factors *R* are based on *F*, with *F* set to zero for negative *F*^2^. The threshold expression of *F*^2^ > σ(*F*^2^) is used only for calculating *R*-factors(gt) *etc*. and is not relevant to the choice of reflections for refinement. *R*-factors based on *F*^2^ are statistically about twice as large as those based on *F*, and *R*- factors based on ALL data will be even larger.

### Fractional atomic coordinates and isotropic or equivalent isotropic displacement parameters (Å^2^)

**Table:**

	*x*	*y*	*z*	*U*_iso_*/*U*_eq_	
N1	0.50055 (17)	0.84855 (17)	0.75856 (18)	0.0503 (5)	
O1	0.29576 (16)	0.32129 (17)	0.56300 (17)	0.0660 (5)	
O2	0.33112 (14)	0.61257 (16)	0.61024 (14)	0.0548 (4)	
H2	0.2948	0.5346	0.5739	0.082*	
O3	0.4312 (2)	0.89120 (17)	0.6494 (2)	0.0793 (5)	
O4	0.55192 (19)	0.93655 (17)	0.85100 (18)	0.0769 (5)	
C1	0.3983 (2)	0.2987 (2)	0.6511 (2)	0.0519 (5)	
H1	0.4261	0.1978	0.6698	0.062*	
C2	0.48004 (18)	0.4189 (2)	0.72888 (18)	0.0391 (4)	
C3	0.44390 (18)	0.5747 (2)	0.70290 (18)	0.0376 (4)	
C4	0.52863 (19)	0.68465 (19)	0.77951 (19)	0.0390 (4)	
C5	0.64236 (19)	0.6430 (2)	0.8776 (2)	0.0440 (5)	
H5	0.6965	0.7184	0.9277	0.053*	
C6	0.67596 (19)	0.4909 (2)	0.9015 (2)	0.0490 (5)	
H6	0.7529	0.4634	0.9669	0.059*	
C7	0.5946 (2)	0.3804 (2)	0.8277 (2)	0.0451 (5)	
H7	0.6168	0.2776	0.8444	0.054*	

### Atomic displacement parameters (Å^2^)

**Table:**

	*U*^11^	*U*^22^	*U*^33^	*U*^12^	*U*^13^	*U*^23^
N1	0.0559 (10)	0.0375 (9)	0.0574 (11)	−0.0026 (7)	−0.0013 (8)	−0.0013 (8)
O1	0.0698 (10)	0.0569 (9)	0.0712 (10)	−0.0092 (7)	−0.0219 (8)	−0.0111 (7)
O2	0.0597 (8)	0.0466 (8)	0.0580 (8)	0.0000 (6)	−0.0271 (7)	0.0031 (6)
O3	0.1053 (13)	0.0440 (9)	0.0886 (12)	0.0024 (8)	−0.0348 (10)	0.0140 (8)
O4	0.1027 (13)	0.0452 (9)	0.0828 (11)	−0.0027 (8)	−0.0148 (9)	−0.0167 (8)
C1	0.0595 (12)	0.0415 (10)	0.0546 (12)	−0.0029 (9)	−0.0042 (10)	−0.0039 (9)
C2	0.0439 (10)	0.0373 (10)	0.0362 (9)	0.0015 (7)	0.0008 (7)	0.0003 (7)
C3	0.0403 (9)	0.0396 (9)	0.0329 (9)	0.0007 (7)	−0.0037 (7)	0.0014 (7)
C4	0.0433 (9)	0.0344 (9)	0.0392 (9)	−0.0012 (7)	−0.0007 (7)	0.0004 (7)
C5	0.0413 (10)	0.0477 (11)	0.0429 (10)	−0.0058 (8)	−0.0039 (8)	−0.0032 (8)
C6	0.0440 (10)	0.0569 (12)	0.0461 (11)	0.0047 (8)	−0.0091 (8)	0.0014 (9)
C7	0.0486 (10)	0.0429 (10)	0.0438 (10)	0.0094 (8)	−0.0007 (8)	0.0036 (8)

### Geometric parameters (Å, °)

**Table:**

N1—O3	1.216 (2)	C2—C3	1.416 (2)
N1—O4	1.220 (2)	C3—C4	1.399 (3)
N1—C4	1.464 (2)	C4—C5	1.384 (2)
O1—C1	1.219 (2)	C5—C6	1.377 (3)
O2—C3	1.340 (2)	C5—H5	0.9300
O2—H2	0.8200	C6—C7	1.374 (3)
C1—C2	1.452 (3)	C6—H6	0.9300
C1—H1	0.9300	C7—H7	0.9300
C2—C7	1.388 (2)		
			
O3—N1—O4	123.06 (18)	C5—C4—C3	121.42 (17)
O3—N1—C4	119.23 (15)	C5—C4—N1	117.45 (15)
O4—N1—C4	117.69 (17)	C3—C4—N1	121.13 (16)
C3—O2—H2	109.5	C6—C5—C4	120.55 (16)
O1—C1—C2	124.42 (19)	C6—C5—H5	119.7
O1—C1—H1	117.8	C4—C5—H5	119.7
C2—C1—H1	117.8	C7—C6—C5	119.31 (17)
C7—C2—C3	120.15 (16)	C7—C6—H6	120.3
C7—C2—C1	119.71 (18)	C5—C6—H6	120.3
C3—C2—C1	120.14 (17)	C6—C7—C2	121.32 (18)
O2—C3—C4	122.28 (16)	C6—C7—H7	119.3
O2—C3—C2	120.47 (16)	C2—C7—H7	119.3
C4—C3—C2	117.24 (16)		
			
O1—C1—C2—C7	179.64 (18)	O3—N1—C4—C5	161.56 (17)
O1—C1—C2—C3	−0.9 (3)	O4—N1—C4—C5	−16.7 (2)
C7—C2—C3—O2	−178.44 (15)	O3—N1—C4—C3	−18.3 (3)
C1—C2—C3—O2	2.1 (2)	O4—N1—C4—C3	163.53 (16)
C7—C2—C3—C4	0.5 (2)	C3—C4—C5—C6	0.5 (3)
C1—C2—C3—C4	−178.95 (15)	N1—C4—C5—C6	−179.32 (16)
O2—C3—C4—C5	178.41 (15)	C4—C5—C6—C7	−0.5 (3)
C2—C3—C4—C5	−0.5 (2)	C5—C6—C7—C2	0.5 (3)
O2—C3—C4—N1	−1.8 (3)	C3—C2—C7—C6	−0.5 (3)
C2—C3—C4—N1	179.32 (14)	C1—C2—C7—C6	178.94 (17)

### Hydrogen-bond geometry (Å, °)

**Table:**

*D*—H···*A*	*D*—H	H···*A*	*D*···*A*	*D*—H···*A*
O2—H2···O1	0.82	1.86	2.597 (3)	148
C5—H5···O2^i^	0.93	2.51	3.422 (4)	168

Symmetry codes: (i) *x*+1/2, −*y*+3/2, *z*+1/2.
